# Comparison of an assumed *versus* measured leucocyte count in parasite density calculations in Papua New Guinean children with uncomplicated malaria

**DOI:** 10.1186/1475-2875-13-145

**Published:** 2014-04-16

**Authors:** Moses Laman, Brioni R Moore, John Benjamin, Nixon Padapu, Nandao Tarongka, Peter Siba, Inoni Betuela, Ivo Mueller, Leanne J Robinson, Timothy M E Davis

**Affiliations:** 1 School of Medicine and Pharmacology, University of Western Australia, Fremantle Hospital, PO Box 480, Fremantle 6959, Western Australia, Australia; 2 Papua New Guinea Institute of Medical Research, Madang, Papua New Guinea; 3 Infection and Immunity Division, Walter and Eliza Hall Institute, Parkville, Victoria, Australia; 4 Center de Recerca en Salut Internacional de Barcelona (CRESIB), Barcelona, Spain

**Keywords:** Malaria, Parasite density, Leucocyte density, *Plasmodium falciparum*, *Plasmodium vivax*

## Abstract

**Background:**

The accuracy of the World Health Organization method of estimating malaria parasite density from thick blood smears by assuming a white blood cell (WBC) count of 8,000/μL has been questioned in several studies. Since epidemiological investigations, anti-malarial efficacy trials and routine laboratory reporting in Papua New Guinea (PNG) have all relied on this approach, its validity was assessed as part of a trial of artemisinin-based combination therapy, which included blood smear microscopy and automated measurement of leucocyte densities on Days 0, 3 and 7.

**Results:**

168 children with uncomplicated malaria (median (inter-quartile range) age 44 (39–47) months) were enrolled, 80.3% with *Plasmodium falciparum* monoinfection, 14.9% with *Plasmodium vivax* monoinfection, and 4.8% with mixed *P. falciparum/P. vivax* infection. All responded to allocated therapy and none had a malaria-positive slide on Day 3. Consistent with a median baseline WBC density of 7.3 (6.5-7.8) × 10^9^/L, there was no significant difference in baseline parasite density between the two methods regardless of *Plasmodium* species. Bland Altman plots showed that, for both species, the mean difference between paired parasite densities calculated from assumed and measured WBC densities was close to zero. At parasite densities <10,000/μL by measured WBC, almost all between-method differences were within the 95% limits of agreement. Above this range, there was increasing scatter but no systematic bias.

****Conclusions**:**

Diagnostic thresholds and parasite clearance assessment in most PNG children with uncomplicated malaria are relatively robust, but accurate estimates of a higher parasitaemia, as a prognostic index, requires formal WBC measurement.

## Background

Although the effect of malaria infection and its treatment on erythrocyte dynamics in humans is well characterized [1,2], there are limited equivalent data on non-erythrocytic haematological indices. Increasing evidence suggests that changes in white blood cell (WBC) densities depend on both the geographical setting and the infecting species of *Plasmodium*[3-6]. An important practical implication of this is for malaria microscopy, which remains the main method of diagnosing malaria and in which a WBC density is required for thick film quantification of parasite density. As well as being a key variable in epidemiological and pharmaceutical intervention trials, the accurate estimation of parasitaemia is important in assessing prognosis in both children [7] and adults [8].

The microscopy method recommended by the World Health Organization (WHO) to estimate asexual parasite density is to multiply the parasite count for a given thick film WBC count (usually 200 or 500) by an assumed peripheral blood WBC count of 8,000/μL [9]. However, some authors have raised concerns regarding its accuracy [5,10-13]. In Papua New Guinea (PNG), epidemiological studies, anti-malarial efficacy trials and routine laboratory reporting have all relied on parasite density calculations based on an assumed WBC density of 8,000/μL regardless of *Plasmodium* species [14-18]. The validity of this approach was assessed by using data collected as part of a trial of artemisinin-based combination therapy in PNG children with uncomplicated malaria in which blood smears were taken and WBC densities measured at study entry and during follow-up as part of efficacy and safety assessment.

## Methods

Children aged 0.5 to 5 years with uncomplicated malaria were recruited at Mugil and Alexishafen Health Centres in Madang Province on the north coast of mainland PNG between April 2011 and June 2012 during a randomized comparative efficacy trial of the artemisinin-based combination therapies artemether-lumefantrine and artemisinin-naphthoquine (Australian New Zealand Clinical Trials Registry ACTRN12610000913077) [19]. Inclusion criteria included: i) an axillary temperature > 37.5°C or fever during the previous 24 hours, ii) *Plasmodium falciparum* (> 1,000 asexual parasites/μL whole blood) and/or *Plasmodium vivax* (> 250/ μL) on a peripheral blood smear, and iii) no clinical or laboratory evidence of severe malaria or other infection according to WHO severity criteria [20]. The study was approved by the PNG Institute of Medical Research Institutional Review Board and the Medical Research Advisory Committee of the PNG Department of Health (MRAC 10.39). Written informed consent was obtained from parents or guardians prior to enrolment of children. All patients were treated with artemisinin-based combination therapy for three days starting on Day 0. Serial clinical assessments were performed by trained research nurses and/or clinicians, and the results documented on standardized case report forms. These assessments included haematological investigations and malaria microscopy on Days 3 and 7.

On Days 0, 3 and 7, venous blood samples were collected into EDTA-containing Microtainer® tubes (Becton, Franklin Lakes, USA) and full blood counts were performed on the same day using a multichannel analyser (ACT diff, Beckman Coulter, Brea, USA). Leucocytosis was defined as a WBC density of ≥ 10.0 × 10^9^/L, leucopaenia as < 4.0 × 10^9^/L and thrombocytopaenia as a platelet density < 100 × 10^9^/L. White blood cell differentials were estimated as percentages with normal lymphocyte, monocyte and granulocyte ranges of 20.5-51.1%, 1.7-9.3% and 42.2-75.2%, respectively.

Of the three blood slides per patient prepared as part of each blood collection, one was stained with 10% v/v Giemsa over 10 minutes and read on site under 100 × magnification by a skilled microscopist to determine patient eligibility. The remaining slides were transported to a reference microscopy laboratory where they were stained with 4% v/v Giemsa stain over 30 minutes and subsequently read independently by two skilled microscopists, who were blinded to the initial result. Parasitaemia was quantified using the numbers of parasites identified in fields containing a total of 200 WBC in high-density smears or 500 WBC in low density smears. Negative slides were those in which no parasites were observed after examination of 200 fields under 100 × magnification. Slides with discrepancies of more than a factor of three regarding density, speciation and/or parasite positivity/negativity were adjudicated by a senior microscopist. Parasite densities were calculated as i) assumed = (parasites counted ÷ number of WBC counted) × 8,000 and ii) confirmed = (parasites counted ÷ number of WBC counted) × (WBC measured by analyser).

Data were analysed using non-parametric methods with a two-tailed significance level of *P* < 0.05. Bland Altman plots were constructed with parasite densities calculated from measured WBC densities used as the gold standard [21].

## Results

Over a 14-month period, 168 children with uncomplicated malaria were enrolled of whom 165 and 164 returned on days 3 and 7, respectively, for follow-up. Their median (inter-quartile range) age was 44 (39–47) months, 49% were males and 55% had a palpable spleen at the time of enrolment. Twenty-five (14.9%) had *P. vivax* monoinfection, 8 (4.8%) had mixed *P. falciparum/P. vivax* infection, while the remaining 80.3% had a *P. falciparum* monoinfection. All patients responded to their allocated therapies and none of the children had a malaria-positive slide on Day 3 of follow-up. There were also significant falls in axillary temperature, pulse rate and respiratory rate during this time (see Table 1). There were no complications related to malaria or its treatment, or deaths (full data to be presented subsequently).

**Table 1:** Clinical and laboratory parameters at baseline and follow-up on Days 3 and 7

	**Day 0 (n = 168)**	**Day 3 (n = 165)**	**Day 7 (n = 164)**	***P *** value*
Axillary Temperature (°C)	38.1 (37.8-38.3)	36.3 (36.2-36.5)	36.6 (36.5-36.6)	< 0.001
Respiratory rate (/min)	32 (30–32)	28 (28–28)	28 (28–28)	< 0.001
Pulse rate (/min)	124 (120–128)	108 (104–109)	104 (103.8-108)	< 0.001
Haemoglobin (g/L)	79 (75–82)	72 (70–75)	78.5 (75–81)	0.003
Total WBC (× 10^9^/L)	7.3 (6.5-7.8)	7.1 (6.6-7.4)	8.3 (7.9-8.7)	< 0.001
Leucopaenia (%)	8.3	4.8	2.4	0.015
Leucocytosis (%)	16.1	7.9	17.7	0.71
Lymphocytes (%)	34 (29–40)	50 (47–52)	45 (43–47)	< 0.001
Monocytes (%)	8 (7–9)	10 (8–11)	8 (6–9)	0.034
Granulocytes (%)	44 (38–46)	32 (30–34)	38 (35–39)	< 0.001
Platelet density (× 10^9^/L)	92 (81–107)	143.5 (130–158)	257 (240–288)	< 0.001
Thrombocytopaenia (%)	53	22.4	3	< 0.001

Data are median and (interquartile range) or percentages.

*Kruskal Wallis test.

Most of the children were anaemic at presentation with a transient further fall in haemoglobin to Day 3 before recovery by Day 7 (see Table 1). The prevalence of leucopenia declined significantly as patients recovered but there was no change in the prevalence of leucocytosis during the one-week follow-up period. More than half of the children had thrombocytopaenia on the day of enrolment but this had dropped to 3% by Day 7. The proportion of children with palpable splenomegaly declined over the week (55% on Day 0 vs 15% on Day 7; *P* < 0.0001).

**Figure 1:**
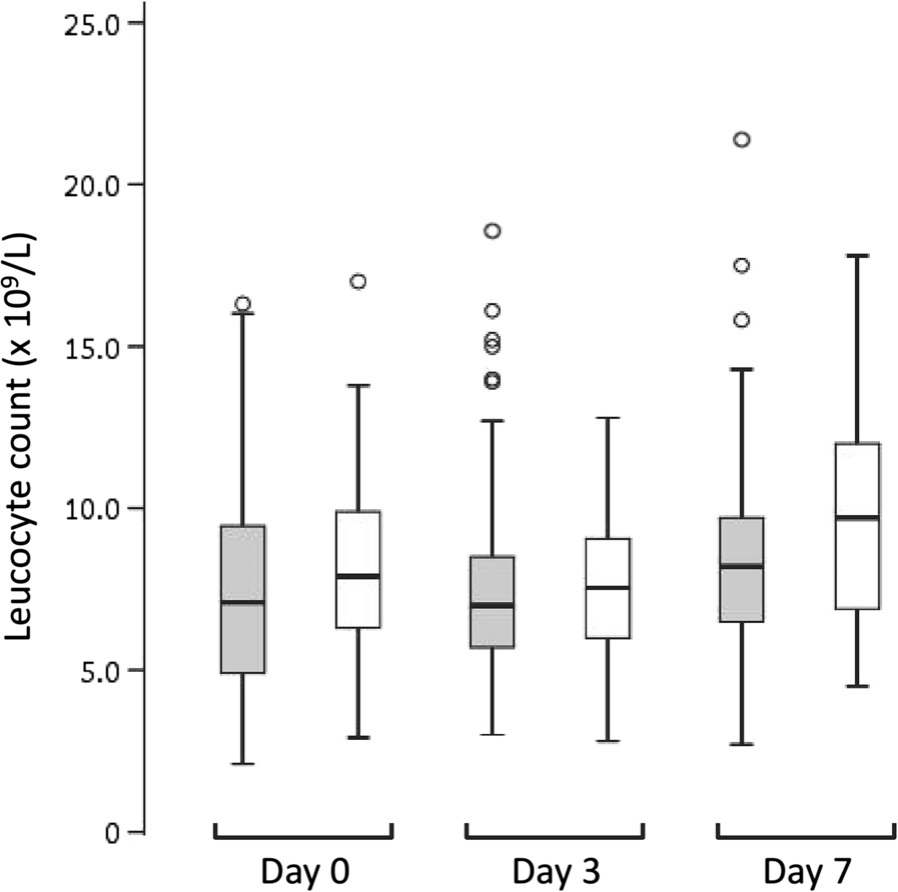
**Box plot showing median, interquartile range, minimum and maximum leucocyte counts in children with falciparum monoinfection (grey boxes) compared to those with vivax monoinfection (white boxes) during the one-week follow-up period.** Outlying values are shown as open circles. There were no significant differences between the two groups on Days 0, 3 and 7 (*P* = 0.18, 0.34 and 0.051, respectively).

The total leucocyte was higher on Day 7 compared to Day 0 (*P* < 0.001) but there was no significant difference between Days 0 and 3 (*P* = 0.81). Excluding those with mixed-species infections, children with falciparum malaria had lower median leucocyte counts at all time-points compared to those with vivax malaria, but the differences were not statistically significant (*P* > 0.05 in each case; Figure 1). In patients with falciparum malaria, 9%, 4.5% and 3% were leucopaenic on Days 0, 3 and 7 respectively, while 6% of children with vivax malaria were leucopaenic on Days 0 and 3, and none were leucopaenic by Day 7. Although there were significant changes in WBC differentials over the course of the one-week period, these changes were all within normal ranges, except for a slightly elevated monocyte count on Day 3 (Table 1).

**Figure 2:**
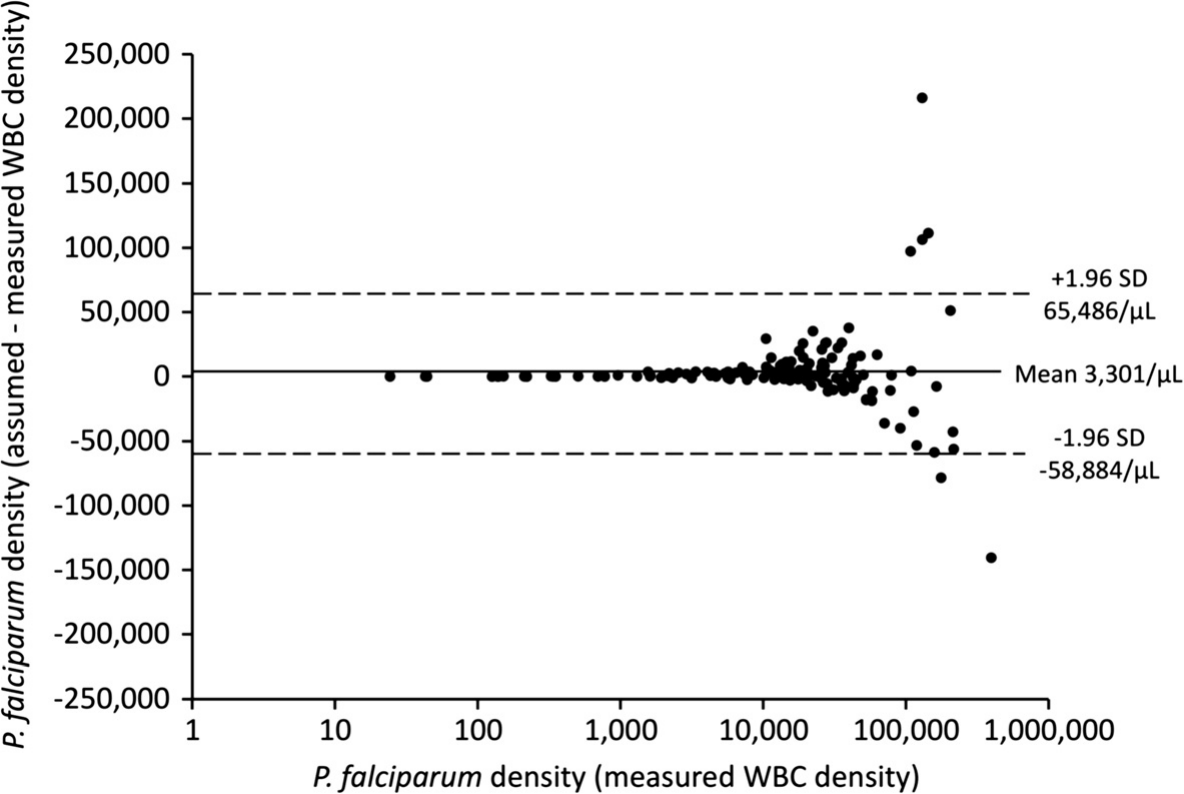
**Bland Altman plot showing parasite densities estimated using measured white blood cell densities on a logarithmic scale for *Plasmodium falciparum *cases (abscissa) and the difference in parasite density from assumed and measured leucocyte densities (ordinate axis).** The mean difference (solid line) and 95% limits of agreement (dashed lines) are also shown.

**Figure 3:**
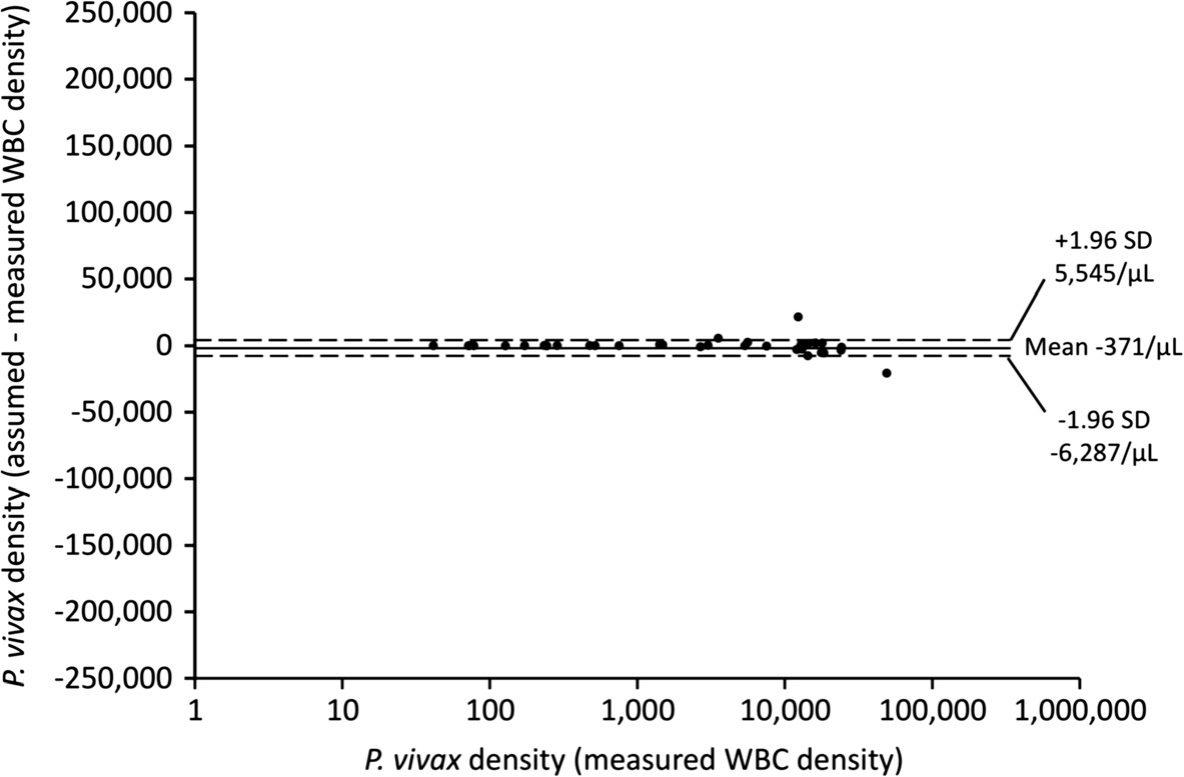
**Bland Altman plot showing parasite densities estimated using measured white blood cell densities on a logarithmic scale for *Plasmodium vivax *cases (abscissa) and the difference in parasite density from assumed and measured leucocyte densities (ordinate axis).** The mean difference (solid line) and 95% limits of agreement (dashed lines) are also shown.

There was no significant difference in baseline parasite density between the two methods regardless of *Plasmodium* species (see Table 2). Bland Altman plots showed that, for both *P. falciparum* and *P. vivax*, the mean difference between paired parasite densities calculated from an assumed and a measured WBC density was close to zero in each case (see Figures 2 and 3). At parasite densities by measured WBC that were < 10,000/μL, and for *P. falciparum* up to 100,000/μL, almost all between-method differences were well within the 95% limits of agreement. At parasite densities above these ranges, there were increasing numbers of co-ordinates outside the 95% limits of agreement. However, there was no pattern to their distribution, indicating that the use of an assumed WBC density of 8,000/μL was not associated with systematic bias, even at the highest parasitaemia.

**Table 2:** Baseline parasite density calculated using an assumed WBC count of 8,000/μL compared to absolute WBC counts determined in individual children

	**Parasite density based on assumed WBC count**	**Parasite density based on measured WBC count**	***P *** value*
*P. falciparum* (n = 143)	15,208 (9,320-19,470)	13,213 (7,680-16,273)	0.49
*P. vivax* (n = 33)	5,364 (644-10,109)	3,538 (595-12,698)	0.96

Data are median (interquartile range). Children with mixed *Plasmodium falciparum/Plasmodium vivax* infections (n = 8) are included in both groups.

*Mann–Whitney U test.

## Discussion

The present study, which is the first of its kind from the Oceania region, shows that parasite densities derived using an assumed WBC of 8,000/μL in PNG children with uncomplicated *P. falciparum* or *P. vivax* infections appear reliable when the parasitaemia is < 10,000/μL. Although there is no systematic bias at higher densities, differences in parasitaemia between assumed and measured WBC densities can be large, especially in *P. falciparum* infections with a parasitaemia > 100,000/μL. The practical implications of these observations are that thresholds for diagnosis and the assessment of parasite clearance in most children with uncomplicated malaria are relatively robust, but that an accurate estimate of higher parasitaemias as a prognostic index in more severely ill children requires formal WBC measurement [9].

Consistent with the overall similarity between the two methods, the median measured total leucocyte density in our patients was close to the assumed 8,000/μL at study entry. A recent study from Ghana suggested that a leucocyte count of 10,000/μL was an appropriate assumed leucocyte count for the determination of parasite density because of significant underestimation associated with 8,000/μL [11]. Although this conclusion was based on a large sample size with pooled data from three separate studies, non-malarial causes of leucocytosis such as bacterial co-infection, which appears relatively common in African settings [22,23], were not excluded. However, in another African study, parasite density based on an assumed leucocyte count of 8,000/μL resulted in a significant overestimation of parasite burden in Ghanaian children [24]. In this study, manual methods were used to quantify WBC and the authors used parametric statistical tests, which may have been inappropriate give the distribution of data. A study of Thai adults with falciparum malaria showed that one-sixth had leucopaenia and that the use of an assumed leucocyte count overestimated parasite densities in nearly one-third of patients [5]. The disparate results of these and the present study highlight the need for local data on WBC densities before the WHO method of quantification of parasitaemia is adopted [9].

Both the African paediatric studies [11,24] were cross-sectional and did not report follow-up WBC counts or clinical outcome data that may have facilitated an assessment of whether malaria was the sole cause of WBC changes at baseline. In the present sample, < 25% of children with strictly defined uncomplicated malaria had either a leucopenia or leukocytosis at presentation and this overall percentage did not change appreciably during the next 7 days based on near-complete follow-up data. This suggests that co-incident infections other than malaria, either ongoing or resolving, did not influence WBC densities in the majority of the present children. In addition, the stability of the WBC densities between Days 0 and 3 suggests that an assumed 8,000/μL could be validly applied for quantitative assessment of parasite clearance which was typically well within 48 hours in the present study.

There was no significant difference in WBC density between children with falciparum malaria and those with vivax malaria in the present study. Some studies have suggested that falciparum malaria is associated with a lower WBC density than vivax malaria [4,5] (consistent with the non-significantly lower medians in children with falciparum malaria in our serial data to Day 7), but others have found no difference [6]. In any case, the absolute difference is of dubious clinical significance [4] especially since a low density may not reflect deficiency but rather localization of leucocytes away from the peripheral circulation in the spleen and other marginal pools [25]. The significant decline in splenomegaly rates between Days 0 and 7 in our children and the parallel normalization of leucocyte counts by Day 7 support this hypothesis.

## Conclusions

The present study shows that *P. falciparum* and *P. vivax* parasite densities estimated using an assumed leucocyte count of 8000/μL are reliable for children without hyperparasitaemia (< 100,000/μL). This is an important and reassuring finding for epidemiological and intervention studies, as well as routine laboratories, in PNG. Although leucocyte densities should ideally be determined in tandem with each thick film in individual patients [26], automated haematological analysers are costly and require disposables, regular maintenance, a reliable power supply and appropriately trained operators. In addition, the manual leucocyte counting method is laborious, subjective and can be inaccurate. The present data confirm that valid parasitaemia data can be collected in situations in PNG, and perhaps other countries with similar malaria epidemiology in the Oceania region, in which there is limited or no capacity to perform manual or automated WBC densities.

## Abbreviations

PNG: Papua New Guinea; WBC: White blood cell; WHO: World Health Organization.

## Competing interests

The authors declare that they have no competing interests.

## Authors’ contributions

All authors contributed to the design of this study, collection of data, analysis of data and/or the interpretation of the results, and to the writing of the manuscript. All authors edited and approved the final version of the manuscript.
